# Use of methenamine hippurate to prevent urinary tract infections in community adult women: a systematic review and meta-analysis

**DOI:** 10.3399/BJGP.2020.0833

**Published:** 2021-05-18

**Authors:** Mina Bakhit, Natalia Krzyzaniak, Joanne Hilder, Justin Clark, Anna Mae Scott, Chris Del Mar

**Affiliations:** Institute for Evidence-Based Healthcare, Bond University, Robina, Australia.; Institute for Evidence-Based Healthcare, Bond University, Robina, Australia.; Allied Health Services, Gold Coast Hospital and Health Services, Robina, Australia.; Institute for Evidence-Based Healthcare, Bond University, Robina, Australia.; Institute for Evidence-Based Healthcare, Bond University, Robina, Australia.; Institute for Evidence-Based Healthcare, Bond University, Robina, Australia.

**Keywords:** antibiotics, methenamine hippurate, primary care, systematic review, urinary tract infections

## Abstract

**Background:**

Urinary tract infections (UTIs) are often treated with antibiotics and are a source of antibiotic overuse.

**Aim:**

To systematically review randomised controlled trials (RCTs) of adult women in the community with a history of recurrent UTIs and who use methenamine hippurate prophylactically.

**Design and setting:**

Systematic review of women in the UK, Australia, Norway, and US (aged ≥18 years) with recurrent UTIs receiving methenamine hippurate against placebo or no treatment, and antibiotics.

**Method:**

The authors searched three databases, clinical trial registries, and performed forward–backward citation analysis on references of included studies.

**Results:**

Six studies involving 557 participants were included (447 were analysed). Of the six studies, five were published and one was an unpublished trial record with results, three compared methenamine hippurate against placebo or control, and three compared methenamine hippurate with antibiotics. For the number of patients who remained asymptomatic, methenamine hippurate showed a non-statistically significant trend of benefit versus antibiotics over 12 months (risk ratio [RR] 0.65, 95% confidence interval [CI] = 0.40 to 1.07, *I*^2^ 49%), versus control over 6 or 12 months (RR 0.56, 95% CI = 0.13 to 2.35, *I*^2^ 93%), and a non-statistically significant trend versus any antibiotic for abacteruria (RR 0.80, 95% CI = 0.62 to 1.03, *I*^2^ 23%). A similar non-statistically significant trend of benefits for methenamine hippurate for the number of UTI or bacteriuric episodes was found, and a non-statistically significant difference in the number of patients experiencing adverse events between methenamine hippurate and any comparator, with a trend towards benefit for the methenamine hippurate, was identified. Antibiotic use and resistance were not consistently reported.

**Conclusion:**

There is insufficient evidence to be certain of the benefits of methenamine hippurate to prevent UTI. Further research is needed to test the drug’s effectiveness in preventing UTIs and as an alternative for antibiotic treatment for UTI.

## INTRODUCTION

Use of antibiotics gives rise to antibiotic resistance, a health crisis that is becoming increasingly critical to address. By 2050, 10 million lives per year and a cumulative 100 trillion USD of economic output will be at risk due to the rise of antibiotic-resistant infections, unless proactive solutions can be found now to slow down the rise of antibiotic resistance.^1^ Conditions often treated with antibiotics and a major source of antibiotic overuse are urinary tract infections (UTIs). UTIs are very common among women, with 50%–60% of women in the US experiencing at least one UTI in their lifetime.^2^

A common dilemma for primary care clinicians is the management of women who have experienced UTIs and re-present with symptoms suggestive of a recurrent UTI. The pain (dysuria), urgency, and frequency associated with recurrent UTIs are highly unpleasant and can disrupt social and occupational activities; all too often, primary care clinicians treat these symptoms — which are suggestive of UTI — by prescribing antibiotics.

If safe, effective treatment alternatives were available, it would be possible to reduce antibiotic prescribing. One possible alternative is methenamine salts, for example, methenamine hippurate, which do not cause antibiotic resistance and act as a bacteriostatic agent through the production of formaldehyde from hexamine in the urine.^3^ Methenamine hippurate is generally safe, and can be used in pregnancy.^4^^,^^5^ It can cause mild gastrointestinal symptoms, and must be used with caution in cases of dehydration, and liver and renal disease.^6^ A previous review of methenamine hippurate compared with no treatment found a significant reduction in UTI symptoms in people without renal tract abnormalities.^7^ However, a comparative effectiveness has not been explored.

The aim of this systematic review was to focus on randomised controlled trials (RCTs) of adult women in the community with a history of recurrent UTIs, who used methenamine hippurate as treatment or prophylaxis.

## METHOD

### Protocol

The review protocol was developed prospectively and registered at the Center for Open Science.^8^ The authors followed the 2-week systematic review processes^9^ and the Preferred Reporting Items for Systematic reviews and Meta-Analyses statement.^10^

### Inclusion and exclusion criteria

Included studies comprised adult women (aged ≥18 years) with a history of recurrent or confirmed UTIs, as defined by study authors, from the community. Studies involving women with spinal cord injuries or those with catheters (long-term or short-term after surgery) were excluded as these groups may experience UTI at different rates from the general population. Studies of males and mixed-gender studies for which separate results for women were not available were excluded.

**Table:** How this fits in

Urinary tract infections (UTIs) are often treated with antibiotics and are a major source of antibiotic overuse. In the interests of decreasing antibiotic prescribing, one alternative treatment is methenamine hippurate, which has reported benefits and a mild side-effect profile; however, its comparative effectiveness has not been widely explored. This study focused on the efficacy of methenamine hippurate compared with both control/no treatment and any antibiotic. Overall, the insufficiency of evidence precludes a firm recommendation on the use of methenamine hippurate prophylactically; however, there is enough evidence to warrant further research to investigate its benefits.

Studies of methenamine hippurate compared with placebo/no treatment or compared with any antibiotic were included; studies that reported the use of an acidifying agent for the urine (for example, ascorbic acid or sodium dihydrogen phosphate) combined with methenamine hippurate were excluded if this was not given to both the control and intervention arms. The primary outcome was UTI manifested by any combination of the following symptoms: dysuria, nocturia, urgency, fever, burning, pyuria, frequency, suprapubic pain, and loin pain. The secondary outcomes were: adverse events, bacteriuria, antibiotic use, and antibiotic resistance.

RCTs of any design (for example, parallel, cluster, or crossover) were included. The authors excluded observational studies and reviews of primary studies (for example, systematic reviews and literature reviews).

### Information sources and search strategy

PubMed, EMBASE, and the Cochrane Central Register of Controlled Trials (CENTRAL) were searched from inception until 13 July 2020. The search string was designed in PubMed, then translated for use in the other databases using the Polyglot Search Translator.^11^ The complete search strings for all databases are provided in Supplementary Box S1.

Clinical trial registries were searched on 13 July 2020 via CENTRAL, which includes the World Health Organization’s International Clinical Trials Registry Platform and clinicaltrials.gov. On 15 July 2020, a citation analysis on the included studies identified by the database searches was conducted; this comprised a backward (cited) analysis, which was conducted manually as the articles were not indexed in Scopus, and a forward (citing) analysis, which was completed using Scopus. The citation analysis was screened against the inclusion criteria.

No restrictions by language or publication date were imposed. Google’s translation services were used to translate the full text of trials that had not been published in English. Included publications were those that were published in full. Publications available as abstract only (for example, conference abstracts) were included if they had a clinical trial registry record, or other public report, with the additional information required for inclusion; publications available as abstract only (for example, conference abstracts) with no additional information available were excluded.

### Screening and data extraction

Three review authors independently screened the titles and abstracts for inclusion against the inclusion criteria. One author retrieved the full texts, and the three authors who screened titles and abstracts screened the full texts for inclusion. Any disagreements were resolved by discussion, or reference to a fourth author.

A data-extraction form was used for study characteristics and outcome data, which was piloted on two studies in the review. Three authors extracted data from the included studies; Box 1 outlines the information that was extracted.

**Box 1.** List of extracted information

**Methods:** study authors, location, study design, and duration of follow-up **Participants:** *n*, age (mean/median, range/SD), number of documented previous UTIs, recent antibiotic use, and whether currently pregnant **Interventions:** methenamine hippurate **Comparators:** placebo/no treatment or any antibiotic **Primary and secondary outcomes:** UTI symptoms, adverse events (such as nausea, diarrhoea, rash, or any symptoms reported as an adverse event by the trial author), bacteriuria, antibiotic use, and antibiotic resistance

*SD = standard deviation. UTI = urinary tract infection.*

### Risk of bias

Three review authors independently assessed the risk of bias for each included study using the Cochrane Risk of Bias Tool (version 1), as outlined in the *Cochrane Handbook for Systematic Reviews of Interventions*.^12^ All disagreements were resolved by discussion or by referring to a fourth author.

### Data synthesis

Review Manager 5 (version 5.4) was used to calculate the treatment effect. Rate ratios were used for results reporting the number of events only, and risk ratios (RRs) or odds ratios (ORs) were used for results reporting the number of patients with an event. The authors planned to use mean differences or standardised mean differences for continuous outcomes if appropriate; however, none of the target outcomes were reported as continuous outcomes in the included studies. Meta-analyses were undertaken when data were sufficient to pool — namely, when two or more studies or comparisons reported the same outcome. A random-effects model was used.

The individual was used as the unit of analysis, when possible; however, if data on the number of individuals with primary and secondary outcomes of interest were not available, the information was extracted as it was presented — for example, the number of events (for example, UTI episodes) in each group. Investigators or study sponsors were contacted to provide missing data, where feasible.

The *I*^2^ statistic was used to measure heterogeneity among the included trials. The authors prespecified that a funnel plot would be created if >10 studies were included; however, <10 studies were included. If data allowed, the following subgroup analyses were prespecified: by comparison, and by duration of intervention. Data were sufficient to conduct a subgroup analysis by comparison (with antibiotic versus with control) only.

It was planned that a sensitivity analysis would be conducted by including, versus excluding, studies with three or more domains rated at high risk of bias; however, as no studies were rated at high risk for three or more domains, that sensitivity analysis was not conducted.

### ‘Summary of findings’ table

One author assessed the quality of evidence using the Grading of Recommendations, Assessment, Development and Evaluations approach,^13^ as recommended by the Cochrane collaboration.^12^ A summary of findings table was created using the following outcomes:

- patients remaining asymptomatic;
- patients remaining abacteriuric;
- number of symptomatic UTI episodes;
- number of bacteriuric UTI episodes; and
- adverse outcomes.

The two comparisons in the summary of findings table were for: methenamine hippurate versus any antibiotic and methenamine hippurate versus control (placebo or antiseptic iodine perineal wash). GRADEpro software was used to prepare the table.

## RESULTS

### Search results

**Figure 1.**
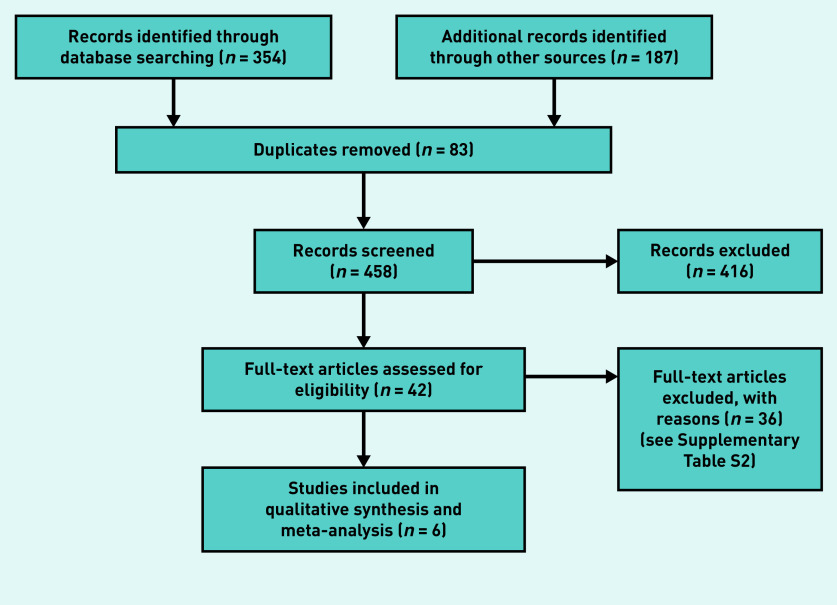
*PRISMA flow diagram.*

The searches across three databases yielded 354 unique records. A backwards (cited) and forwards (citing) citation analysis yielded an additional 176 records, and the clinical registry search returned 11 records; after deduplication, this resulted in a total of 458 records to screen. In total, 416 records were excluded after screening the title and abstract; 42 records were obtained for full-text screening, which included 41 references from the database searches and one clinical registry record that included results information. Supplementary Table S1 outlines eligible ongoing studies with no published or reported results. After full-text screening, 36 references were excluded; the characteristics of excluded studies are given in Supplementary Table S2. Six studies were included in the qualitative synthesis and meta-analysis (Figure 1).^5^^,^^14^^–^^18^

### Risk of bias

**Figure 2.**
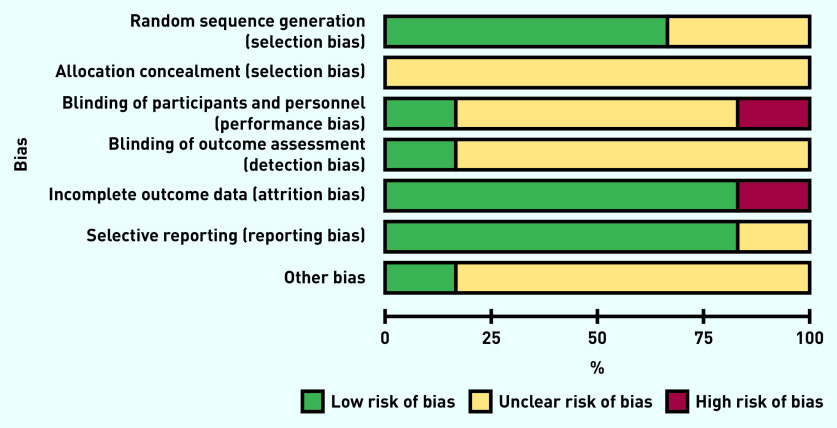
*Risk of bias graph: review authors’ judgements about each risk of bias item presented as percentages across all included studies.*

Due to the age of the included studies (five of the six included studies were >30 years old), the overall risk of bias was generally unclear. When these studies were published, reporting guidelines were not routinely used, which had an impact on the quality and degree of information reported: notably, sequence generation, allocation concealment, and blinding (both of participants and personnel, and of outcome assessment) were unclear (Figure 2). No evidence of incomplete outcome data or selective reporting of outcomes was found. The authors’ conflicts of interest and study funding were also inadequately described in most trials.

### Characteristics of included studies

Five of the included studies were published RCTs,^5^^,^^14^^–^^17^ and one was a clinical trial registry record with results provided.^18^ All were parallel RCTs, evenly split between two-arm and three-arm trials. Two studies were published in Norwegian;^16^^,^^17^ the remaining four were published in English.^5^^,^^14^^,^^15^^,^^18^ Characteristics of all six studies included in the review are given in Table 1.

**Table 1.** Characteristics of studies included in review

**Study authors (year)**	**Country**	**RCT type**	**Randomised participants, *n* (mean age, years)**	**Participants analysed, *n***	**Previously documented UTIs per unit of time, *n***	**Intervention, dose, frequency, duration**	**Comparator, dose, frequency**	**Duration of follow-up**
Brumfitt *et al* (1981)^14^	UK	Two-arm, parallel	110 (intervention: 35.9; comparator: 31.3)	99 (intervention, *n* = 56; comparator, *n* = 43)	Intervention: mean 6.5 per 12 months; comparator: mean 6.6 per 12 months	Intervention: methenamine hippurate, 1 g, twice daily, 12 months	Comparator: nitrofurantoin, 50 mg, twice daily	12 months
Brumfitt *et al* (1983)^15^	UK	Three-arm, parallel	67 (intervention: 38.2; comparator 1: 39.9; comparator 2: 31.7)	64 (intervention, *n* = 25; comparator 1, *n* = 20; comparator 2, *n* = 19)	Intervention: mean 4.9 per 12 months; comparator 1: mean 6.4 per 12 months; comparator 2: mean 5.2 per 12 months	Intervention: methenamine hippurate, 1 g, twice daily, 12 months	Comparator 1: trimethoprim, 100 mg, once nightly; comparator 2: povidone iodine, 10% solution diluted 15 mL to 240 mL water/antiseptic perineal wash, minimum twice daily	12 months
Furness *et al* (1975)^5^	Australia	Three-arm, parallel	206 (all of childbearing age)	206 (intervention 1, *n* = 70; intervention 2, *n* = 69; comparator, *n* = 67)	NR	Intervention 1: methenamine hippurate, 1 g, twice daily, NR; intervention 2: methenamine mandelate, 1 g, four times daily, NR	Control: no treatment	24 months^a^
Gundersen *et al* (1986)^16^	Norway	Two-arm, parallel	30 (intervention: 74.5; comparator: 74.0)	30 (intervention, *n* = 15; comparator, *n* = 15)	At least two in the previous 6 months	Intervention: methenamine hippurate, 1 tablet (dose NR), twice daily, 6 months	Placebo: one tablet, twice daily	6 months
Høivik *et al* (1984)^17^	Norway	Three-arm, cluster	52 (intervention 1: 19.1; intervention 2: 28.9; comparator: 29.3)	52 (intervention 1, *n* = 28; intervention 2, *n* = 12; comparator, *n* = 12)	Intervention 1: mean 3.6 per 12 months; intervention 2: mean 4.1 per 12 months; comparator: mean 3.8 per 12 months	Intervention 1: methenamine hippurate, 1 tablet (dose NR), twice daily, 12 months; intervention 2: methenamine hippurate, 1 tablet (dose NR), once nightly, with one placebo tablet per 12 months	Placebo: two tablets, twice daily	12 months
Botros^b^ (2020)^18^	US	Two-arm, parallel	92 (intervention: 70; comparator: 73)	86 (intervention, *n* = 43; comparator, *n* = 43)	NR	Intervention: methenamine hippurate (dose NR)	Comparator: trimethoprim (dose NR)	12 months

a *This is the reported duration of the whole trial; however, the authors do not state the duration of the intervention.*

b *Data extracted from the clinical trial registry record. NR = not reported. RCT = randomised controlled trial. UTI = urinary tract infection.*

A total of 557 participants were included in the trials, of which 447 were analysed; only one study^16^ involved <50 participants. Three studies compared methenamine hippurate with a placebo or control, and three compared the efficacy of methenamine hippurate with antibiotics — namely, trimethoprim (two trials^15^^,^^18^) and nitrofurantoin (one trial^14^). One RCT^15^ examined a second non-antibiotic comparator, povidone-iodine solution, a common antiseptic perineal wash.

### Prevention of UTI

#### Patients remaining asymptomatic after 6 or 12 months

**Figure 3.**
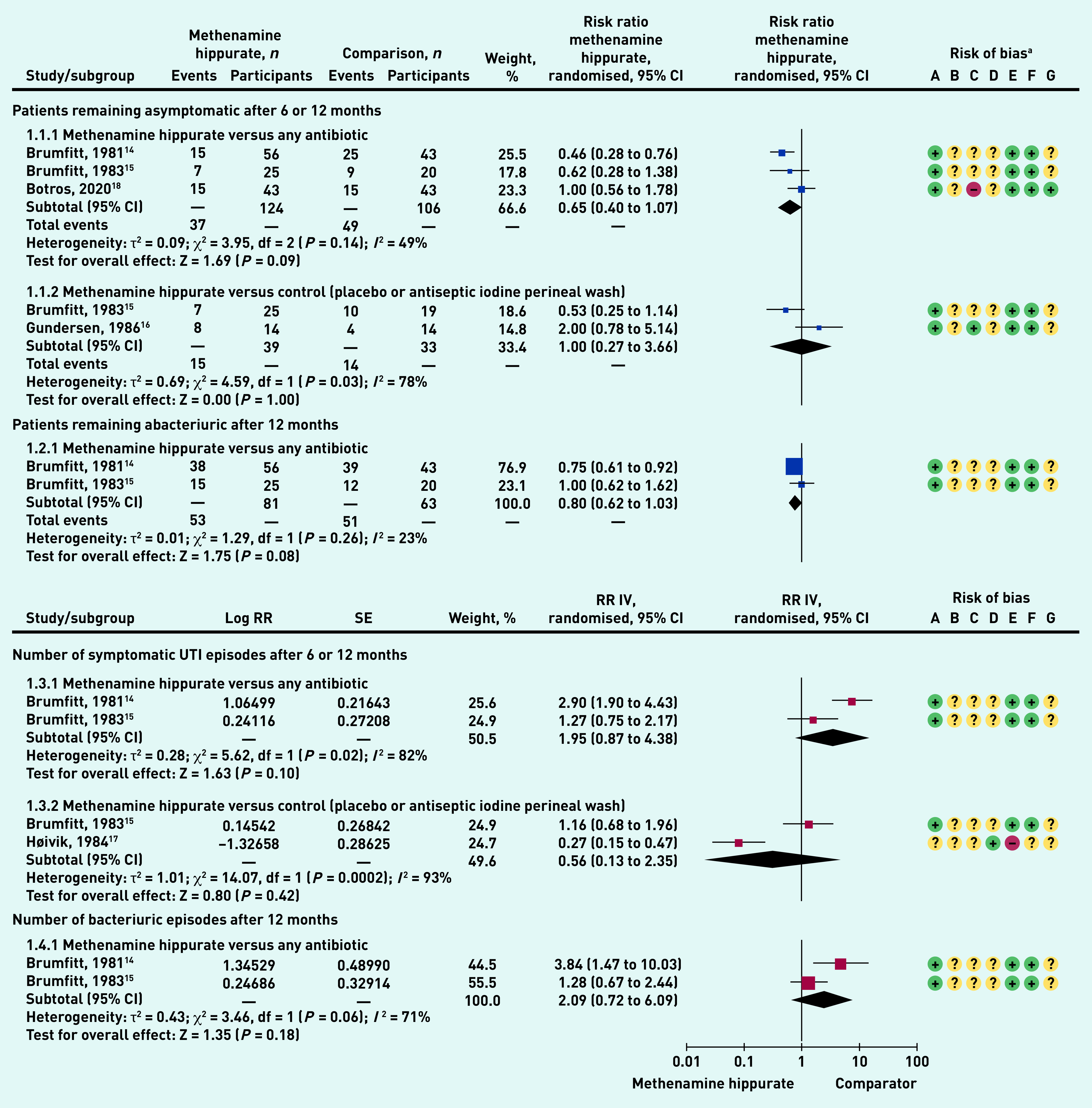
*Prevention of UTI meta-analysed outcomes.* ^a^ *Risk of bias: A = random sequence generation (selection bias); B = allocation concealment (selection bias); C = blinding of participants and personnel (performance bias); D = blinding of outcome assessment (detection bias); E = incomplete outcome data (attrition bias); F = selective reporting (reporting bias); and G = other bias. df = degrees of freedom. CI = confidence interval. IV = inverse variance. Log = natural logarithm. RR = rate ratio. SE = standard error. UTI = urinary tract infection.*

Methenamine hippurate versus antibiotics showed a non-statistically significant trend of benefit for methenamine hippurate (RR 0.65, 95% confidence interval [CI] = 0.40 to 1.07), and heterogeneity was moderate (49%). Methenamine hippurate versus control (placebo or antiseptic iodine perineal wash) showed a non-statistically significant difference between groups (RR 1.0, 95% CI = 0.27 to 3.66), and heterogeneity was high (78%) (Figure 3).

#### Patients remaining abacteriuric after 12 months

Methenamine hippurate versus any antibiotic showed a trend to benefit for methenamine hippurate, but a non-statistically significant difference (RR 0.80, 95% CI = 0.62 to 1.03), with low heterogeneity (23%) (Figure 3).

#### Number of symptomatic UTI episodes after 6 or 12 months

Methenamine hippurate versus any antibiotic showed a trend to benefit for methenamine hippurate, but no statistically significant difference (RR 1.95, 95% CI = 0.87 to 4.38). Heterogeneity was high (82%). Methenamine hippurate versus control (placebo or antiseptic iodine perineal wash) showed a trend to benefit for methenamine hippurate, but no statistically significant difference between groups (RR 0.56, 95% CI = 0.13 to 2.35). Heterogeneity was high (93%) (Figure 3).

#### Number of bacteriuric episodes after 12 months.

Methenamine hippurate versus any antibiotic showed a trend to benefit for methenamine hippurate, but no statistically significant difference between groups (RR 2.09, 95% = CI 0.72 to 6.09), with high heterogeneity (71%) (Figure 3). One study^5^ reported on the number of patients with post-natal bacteriuria, finding no significant difference between methenamine hippurate, methenamine mandelate, and no-treatment groups (data not shown).

### Adverse events

**Figure 4.**
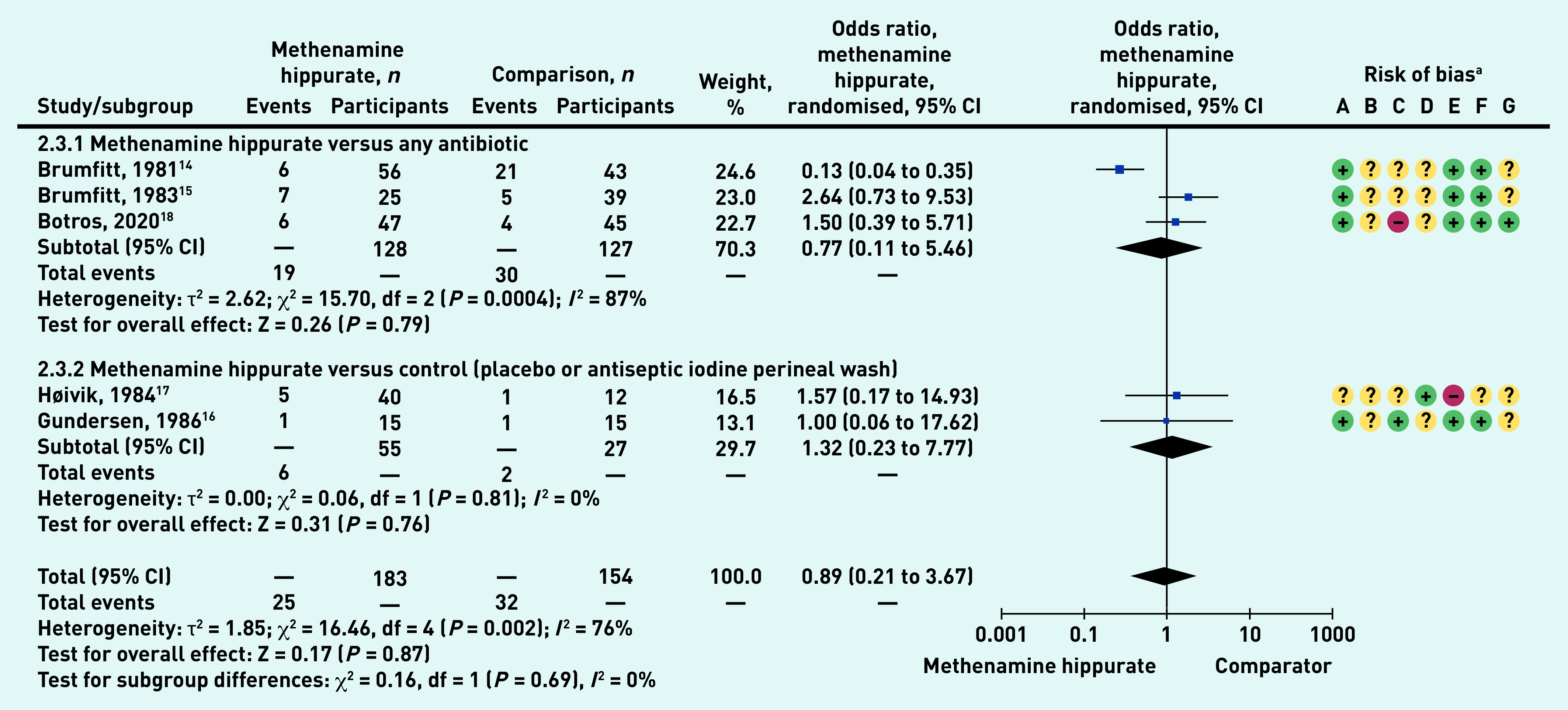
*Number of patients with adverse outcomes: methenamine hippurate versus comparator – sub-grouped by comparator type (any antibiotic, control).* *^a^Risk of bias: A = random sequence generation (selection bias); B = allocation concealment (selection bias); C = blinding of participants and personnel (performance bias); D = blinding of outcome assessment (detection bias); E = incomplete outcome data (attrition bias); F = selective reporting (reporting bias); and G = other bias. CI = confidence interval. df = degrees of freedom.*

The most common adverse events reported in all studies^5^^,^^14^^–^^18^ were nausea, headache, and abdominal pain (data not shown). There was no statistically significant difference in the number of patients experiencing any adverse events when methenamine hippurate was compared with any antibiotic (odds ratio [OR] 0.77, 95% CI = 0.11 to 5.46), or with control (OR 1.32, 95% CI 0.23 to 7.77) (Figure 4). There was also no overall difference between methenamine hippurate and any comparator (total OR 0.89, 95% CI = 0.21 to 3.67).

### Antibiotic use

The use of antibiotics outside of an intervention was indirectly reported in three trials;^5^^,^^16^^,^^17^ in each, antimicrobials were utilised to treat those participants who experienced a recurrent UTI during the trial (data not shown).

Two studies^16^^,^^17^ reported that, in the case of symptoms of UTI and a positive bacteriological urine test, the intervention/comparator was ceased; antibiotic therapy was initiated and used until sterile urine was achieved, whereby prophylactic treatment was resumed. One study^16^ reported seven symptomatic UTI recurrences in the methenamine hippurate group and 29 in the placebo group, each of which might have required the initiation of antibiotics. Similarly, another study^17^ reported 19 recurrences in the group receiving 2 g methenamine hippurate compared with four recurrences in the arm receiving a dose of 1 g and 26 recurrences in the placebo group (data not shown).

One study^5^ specified that patients received antibiotics on developing clinical pyelonephritis: 20% of participants receiving methenamine hippurate, 13% of those receiving methenamine mandelate, and 25% of those in the control group were diagnosed with pyelonephritis (data not shown).

### Antibiotic resistance

Antibiotic resistance was poorly reported among the included studies as only three RCTs investigated sensitivity patterns of bacteria causing the infection.^14^^,^^15^^,^^18^ In one trial,^14^ authors reported that 38% of recurrent UTIs were caused by resistant strains; however, they did not report the between group difference. In another trial,^15^ authors reported that 82% of the isolated strains were resistant to the intervention antibiotic arm (trimethoprim) compared with 3% in the methenamine hippurate arm. Authors in the third trial^18^ reported a higher number of resistant strains identified in the methenamine hippurate arm (*n* = 58) compared with the antibiotic arm (*n* = 30); however, no additional data or explanations were provided to explain the difference (see Supplementary Table S3).

### Adherence

Although all studies reported that methenamine hippurate was used for a minimum duration of 6 months, which may raise concerns about adherence to the prophylaxis, some data suggests this may be acceptable to patients. For example, one study^14^ found a mean longer adherence to therapy for methenamine hippurate (196 days) than for nitrofurantoin (66 days), and another^18^ suggests a mean higher adherence in the group receiving methenamine hippurate than in the group receiving trimethoprim, when measured on the eight-item Morisky Medication Adherence Scale (data not shown).

### Summary of findings tables

Table 2 shows the summary of findings table for methenamine hippurate versus any antibiotic; Table 3 shows the summary of findings table for methenamine hippurate versus control.

**Table 2.** Summary of findings: methenamine hippurate versus any antibiotic for the prevention of UTI^a^

**Certainty assessment**							**Patients, *n***		**Effect**		**Certainty^b^**
**Studies, *n***	**Study design**	**Risk of bias**	**Inconsistency**	**Indirectness**	**Imprecision**	**Other considerations**	**Methenamine hippurate, *n* (%)**	**Any antibiotic, *n* (%)**	**Relative RR (95% CI)**	**Absolute RR (95% CI)**	
Patients remaining asymptomatic (follow-up: 6–12 months; assessed with number of patients that remained asymptomatic)											
3	RCT	Serious^c^	Serious^d^	Not serious	Serious^e^	Publication bias strongly suspected^f^	37/124 (29.8)	49/106 (46.2)	0.65 (0.40 to 1.07)	162 fewer per 1000 (from 277 fewer to 32 more)	Very low
Patients remaining abacteriuric (follow-up: 12 months; assessed with number of patients that remained abacteriuric)											
2	RCT	Serious^c^	Not serious	Not serious	Serious^e^	Publication bias strongly suspected^f^	53/81 (65.4)	51/63 (81.0)	0.80 (0.62 to 1.03)	162 fewer per 1000 (from 308 fewer to 24 more)	Very low
Symptomatic UTI episodes (follow-up: 6–12 months; assessed with number of symptomatic UTI episodes)											
2	RCT	Serious^c^	Not serious	Serious^g^	Serious^h^	Publication bias strongly suspected^f^	The pooled results of two RCTs on the number of symptomatic UTI episodes showed a trend of benefit for methenamine hippurate with a total RR of 1.95 (95% CI = 0.87 to 4.38) but the result was statistically insigificant with high heterogeneity (*I*^2^ 82%)				Very low
Bacteriuric UTI episodes (follow-up: 12 months; assessed with number of bacteriuric UTI episodes)											
2	RCT	Serious^c^	Not serious	Serious^g^	Serious^h^	Publication bias strongly suspected^f^	The pooled results of two RCTs on the number of bacteriuric UTI episodes showed a non-statistically significant trend of benefit for methenamine hippurate with a total RR of 2.09 (95% CI = 0.72 to 6.09) with high heterogeneity (*I*^2^ 71%)				Very low
Any adverse outcomes (follow-up: 12 months; assessed with number of patients with a reported adverse outcome)											
3	RCT	Serious^c^	Serious^d^	Not serious	Serious^e^	Publication bias strongly suspected ^f^	19/128 (14.8)	30/127 (23.6)	0.77 (0.11 to 5.46)	54 fewer per 1000 (from 210 fewer to 1000 more)	Very low

a *Setting: community, outpatient, and primary care.*

b *GRADE Working Group grades of evidence — high: very confident that the true effect lies close to that of the estimate of the effect; moderate: moderately confident in the effect estimate — the true effect is likely to be close to the estimate of the effect but there is a possibllity that it is substantially different; low: confidence in the effect estimate is limited — the true effect may be substantially different from the estimate of the effect; very low: little confidence in the effect estimate — the true effect is likely to be substantially different from the estimate of effect.*

c *Allocation concealment and blinding of outcome assessment is unclear.*

d *Unexplained heterogeneity. In Botros 2020: hippurate dose is not reported and could be different from the other included studies.*

e *Small sample size.*

f *Small number of studies hindered assessment of publication bias.*

g *High heterogeneity.*

h *Small number of events occurring in both groups. CI = confidence interval. RCT = randomised controlled trial. RR = risk ratio. UTI = urinary tract infection.*

**Table 3.** Summary of findings: methenamine hippurate versus control (placebo or antiseptic iodine perineal wash) for the prevention of UTI^a^

**Certainty assessment**							**Patients, *n***		**Effect**		**Certainty^b^**
**Studies, *n***	**Study design**	**Risk of bias**	**Inconsistency**	**Indirectness**	**Imprecision**	**Other considerations**	**Methenamine hippurate, *n* (%)**	**Control, *n* (%)**	**Relative RR (95% CI)**	**Absolute RR (95% CI)**	
Patients remaining asymptomatic (follow-up: 6–12 months; assessed with number of patients that remained asymptomatic)											
2	RCT	Serious^c^	Very serious^d^	Not serious	Serious^e^	Publication bias strongly suspected ^f^	15/39 (38.5)	14/33 (42.4)	1.00 (0.27 to 3.66)	0 fewer per 1000 (from 310 fewer to 1000 more)	Very low
Symptomatic UTI episodes (follow-up: 12 months; assessed with number of symptomatic UTI episodes)											
2	RCT	Serious^c^	Serious^g^	Not serious	Serious^e^	Publication bias strongly suspected^f^	The pooled results of two RCTs on the number of symptomatic UTI episodes showed a non-statistically significant trend of benefit for methenamine hippurate with a total RR of 0.56 (95% CI = 0.13 to 2.35) with high heterogeneity (*I*^2^ 93%)				Very low
Any adverse outcomes (follow-up: 12 months; assessed with number of patients with a reported adverse outcome)											
2	RCT	Serious^c^	Not serious	Not serious	Serious^e^	Publication bias strongly suspected ^f^	6/55 (10.9)	2/27 (7.4)	1.32 (0.23 to 7.77)	24 more per 1000 (from 57 fewer to 501 more)	Very low

a *Setting: community, outpatient, and primary care.*

b *GRADE Working Group grades of evidence — high: very confident that the true effect lies close to that of the estimate of the effect; moderate: moderately confident in the effect estimate — the true effect is likely to be close to the estimate of the effect but there is a possibllity that it is substantially different; low: confidence in the effect estimate is limited — the true effect may be substantially different from the estimate of the effect; very low: little confidence in the effect estimate — the true effect is likely to be substantially different from the estimate of effect.*

c *Allocation concealment and blindind of outcome assessment is unclear.*

d *High heterogeneity as each study compared methenamine hippurate against a different control group.*

e *Small sample size.*

f *Small number of studies hindered assessment of publication bias.*

g *High heterogeneity. CI = confidence interval. RCT = randomised controlled trial. RR = risk ratio. UTI = urinary tract infection.*

## DISCUSSION

### Summary

Six studies evaluating oral methenamine hippurate for preventing UTI in women with recurrent UTI were identified. Included studies showed a trend towards the benefit of methenamine hippurate for use to prevent recurrent UTI, although meta-analyses showed no statistically significant differences between methenamine hippurate and any comparators.

### Strengths and limitations

This review provides rigour by excluding studies at high risk of bias due to confounding variables (such as post-surgery preventive studies and those involving women with indwelling catheters), thereby rendering the results more relevant to women in the community.

There are limitations, however: only a small number of studies were included and there are several reasons why the findings of the individual studies should be interpreted with caution. Five of the studies^5^^,^^14^^–^^17^ were published >30 years ago and the most recent study^18^ was a clinical trial record, not a peer-reviewed publication. The included studies also featured: considerable clinical and statistical heterogeneity; poor reporting of bacterial resistance as one of the harms of using antibiotics in trials with an antibiotic arm; and general unclear risk of bias. In addition, only one study reported the use of methenamine mandelate and the sample size was small,^5^ hindering the reviewers from including methenamine mandelate in the analysis. This constitutes a deviation from the protocol, which specified that subgroup analyses and sensitivity analyses would be conducted; these could not be carried out because of the paucity of available data.

### Comparison with existing literature

A Cochrane systematic review previously assessed methenamine hippurate for prevention of UTIs.^7^ It included studies with the following characteristics: RCTs and quasi-RCTs, looking at all population groups, and comparing intervention to control/no treatment only. The review showed some benefit for methenamine hippurate for preventing UTIs. The review presented here differs in that it only includes studies that were RCTs (the clinical trial record was an unpublished RCT study), comparing intervention to both control/no treatment and to any antibiotic (as this is one of the indications where antibiotics are routinely prescribed for a long duration). Moreover, it also focuses on women in the community, excluding studies with any male participants and those including participants at high risk of UTI infections (that is, those with abnormal renal tract anatomy, those who are immunocompromised, or those who have spinal cord injuries), which improves the applicability of the results to the majority of UTI cases seen in the community.

Similarly to the Cochrane review, the authors also found an overall low rate of adverse events, which suggests that methenamine hippurate is unlikely to be causing any harms; however, there is a need to explore this further in larger RCTs as methenamine hippurate, as a treatment, must be taken frequently over a long period of time (up to 12 months).

The authors are aware of an ongoing study that investigates prophylaxis methenamine hippurate use against antibiotic;^19^ however, the data were not available.

### Implications for research and practice

Methodological and reporting limitations, as well as the small volume of available evidence, preclude a firm recommendation about the use of methenamine hippurate for prophylaxis in women with recurrent UTIs. The minimal reporting of harms from methenamine hippurate, along with the trend towards its benefit in reducing recurrent UTI, suggests a possible avenue to further investigate methenamine salts’ benefits for preventing recurrent UTIs.
